# Smartphone Apps for Vaping Cessation: Quality Assessment and Content Analysis

**DOI:** 10.2196/31309

**Published:** 2022-03-28

**Authors:** Sherald Sanchez, Anasua Kundu, Elizabeth Limanto, Peter Selby, Neill Bruce Baskerville, Michael Chaiton

**Affiliations:** 1 Institute of Medical Science Termerty Faculty of Medicine University of Toronto Toronto, ON Canada; 2 Ontario Tobacco Research Unit Dalla Lana School of Public Health University of Toronto Toronto, ON Canada; 3 Department of Pharmacology & Toxicology University of Toronto Toronto, ON Canada; 4 Nicotine Dependence Service Centre for Addiction and Mental Health Toronto, ON Canada; 5 Department of Family and Community Medicine University of Toronto Toronto, ON Canada; 6 Office of Audit and Evaluation Canadian Institutes of Health Research Ottawa, ON Canada; 7 Institute for Mental Health Policy Research Centre for Addiction and Mental Health Toronto, ON Canada

**Keywords:** e-cigarettes, vaping, cessation, mHealth interventions

## Abstract

**Background:**

As the prevalence of electronic cigarette (e-cigarette) use, or vaping, continues to grow, particularly among young people, so does the need for research and interventions to address vaping.

**Objective:**

This study examines the quality of free vaping cessation apps, their contents and features, popularity among users, and adherence to evidence-based principles.

**Methods:**

A systematic search of existing apps for vaping cessation was conducted in December 2020. Eligible apps were free, in English, and included features specifically targeting vaping cessation. Each app included in the analysis was used daily for at least seven consecutive days, assessed using the Mobile App Rating Scale, and rated by at least two authors (AK, EL, or SS) based on adherence to evidence-based practices. Intraclass correlation coefficient (ICC) estimates were computed to assess interrater reliability (excellent agreement; ICC 0.92; 95% CI 0.78-0.98).

**Results:**

A total of 8 apps were included in the quality assessment and content analysis: 3 were developed specifically for vaping cessation and 5 focused on smoking cessation while also claiming to address vaping cessation. The mean of app quality total scores was 3.66 out of 5. Existing vaping cessation apps employ similar approaches to smoking cessation apps. However, they are very low in number and have limited features developed specifically for vaping cessation.

**Conclusions:**

Given the lack of vaping cessation interventions at a time when they are urgently needed, smartphone apps are potentially valuable tools. Therefore, it is recommended that these apps apply evidence-based practices and undergo rigorous evaluations that can assess their quality, contents and features, and popularity among users. Through this process, we can improve our understanding of how apps can be effective in helping users quit vaping.

## Introduction

In 2020, the US Surgeon General’s *Report on Smoking Cessation* identified the development of interventions to address vaping, or electronic cigarette (e-cigarette) use, particularly among young people, as a research priority [1]. There is “substantial evidence” [2] that vaping can lead to nicotine dependence and growing evidence that dependence symptoms are increasing among young people [3-8]. Several studies have shown that many youth are making attempts to quit, which are often unsuccessful due to the difficulty of quitting unassisted [5-9]. Help-seeking youth need access to tools and services that can aid them in their quitting journey. However, the availability of vaping cessation interventions remains limited in huge part because our understanding of the process of vaping cessation is extremely limited and evidence-based guidelines and interventions have yet to be developed.

Mobile software apps are highly accessible and cost-effective platforms for interventions that can be customized by the user and provide real-time support. The use of apps to provide support for addressing substance use, such as alcohol and tobacco, is well-documented in the literature [10-16]. Among young people who are the top smartphone users [17] globally and a high-risk group for the public health consequences of e-cigarettes [2,18], smartphone apps are a potential way to offer support and promote successful quit attempts among help-seeking e-cigarette users, or vapers. Informal searches revealed a handful of apps for vaping cessation currently available. However, little is known about their quality and the degree to which their contents reflect the current state of evidence [9]. Accordingly, this study examined the quality of free vaping cessation apps, their contents and features, popularity among users, and adherence to evidence-based practices. To our knowledge, this study was the first systematic analysis of free apps for vaping cessation. We focused on apps that were in English and free to download. We also decided to only include apps that were available in both the Apple App Store and Google Play Store at the time of the review. There are 2 key reasons for this. First, at the time, a study in the *Journal of Medical Internet Research* found that 87% of vaping-related apps in the Google Play Store were aimed at assisting users to sustain their vaping and improve their experience of vaping, including informational apps with “recipes” for vapers who want to mix their own vape liquids, retail apps for ordering vaping paraphernalia online, or navigation apps for locating vape stores nearby. The same study found that only 3% had features to help users quit [19]. By contrast, Apple implemented a ban on apps promoting recreational vaping from its online market in response to a recent outbreak of vaping-related lung injuries [20]. Second, compared with Google, Apple has a more rigorous and transparent review process for apps, which is described in a designated developer web page. We decided to use these resources to our advantage in conducting this review.

## Methods

### Search Strategy

Using the terms *vaping*, *vaping cessation*, *quit vaping*, *stop vaping*, and *no vaping*, we searched for smartphone apps targeting vaping cessation on the Canadian Apple and Google online stores in December 2020. Preliminary searches on the Apple App Store website and the embedded App Store app in iPhones showed drastically different sets of results. Whereas the phone search yielded more than 185 apps, the website search using the same strategy showed between 6 and 10 apps. For consistency with the average user experience, we decided to conduct the formal searches using the phone app instead of the website.

### Eligibility Criteria

Apps that were not in English and not free to download were excluded in the preliminary screening. As previously noted, apps that were not available in both the Google Play Store and the Apple App Store were excluded. Based on informal searches conducted prior to the review (AK, EL, and SS), we expected our search terms to yield a large number of apps for smoking cessation. Our approach to these apps was to check the descriptions and profiles of popular smoking cessation apps to confirm whether they also addressed vaping cessation. Those that did not were excluded at this stage. Three authors (AK, EL, and SS) independently reviewed the remaining eligible apps (n=39) through a 2-pronged screening approach: first, we examined their profiles on the online app stores; then, if the information was available, we did an ancestry search of developer websites, profiles, and overall online presence. From this, those that were confirmed to have no association with vaping cessation were excluded at this stage. A total of 9 apps were downloaded as part of the final sample.

### Assessment of Quality, Contents and Features, and Popularity Among Users

Each app was used daily for at least seven consecutive days, which informed the assessment of quality, analysis of app contents and features, and classification of apps. To estimate popularity among users, number of downloads was used as an indicator. Given that information on app downloads is only available for the apps in the Google Play Store, user ratings and number of reviews were used as a secondary measure of popularity.

The quality of each app was assessed using the Mobile App Rating Scale (MARS), a multidimensional measure for rating the quality of mobile health apps [21]. The MARS consists of 5 categories—engagement, functionality, aesthetics, information quality, and subjective quality—with 23 collective items rated using a 5-point scale. An option of “N/A” or “not applicable” is available for items that cannot be adequately assessed. Subjective quality is an optional category; thus, it was excluded from our scoring. Mean scores for each of the 4 objective categories were calculated to identify strengths and weaknesses of each app, while the sum of all 4 scores provided an overall quality score for each app. Given the variability found among health-related apps, MARS itself does not prescribe a defined threshold for the type of scores a high-quality app should obtain on the scale [21]. However, a previous study [15] that assessed the quality and content of smoking cessation apps identified a MARS total mean score of 3 as the cut-off for apps with acceptable quality.

Based on our analysis of app contents and features, each app was classified according to its primary approach to supporting vaping cessation. We followed Abroms et al’s [11,12] categories for smoking cessation apps, which included “calculators” tracking money saved and health benefits gained since quitting; “calendars” monitoring number of days before or after the quit date; and “informational content” providing general information on quitting. Informational content on each app was cross-referenced with the current state of evidence on the health consequences of vaping [2].

### Adherence to Evidence-Based Practices

To assess clinical quality, previous studies of a similar design evaluated apps based on their level of adherence to clinical practice guidelines [11-15]. In this study, we developed a 14-item Adherence Index based on a modified version of the *Canadian Smoking Cessation Clinical Practice Guideline* [22] with the support of an experienced clinician who led the development of the guideline on smoking cessation (PS). These 14 items are shown in Table 1. The items are written in plain language. Most of the content remained the same, with 1 key change—because there is currently no strong evidence supporting use of nicotine replacement therapy for vaping cessation, endorsement of medication was omitted. The process of modifying the guidelines was further informed by earlier studies in identifying key differences between smoking and vaping based on the experiences of help-seeking youth and young adult vapers [8,23]. Each item on the Adherence Index was coded *present* or *not present*.

**Table 1:** Characteristics of vaping cessation apps and summary of quality scores.

Characteristics		Kwit	Quit Vaping - For Good	Quit Vaping Addiction Calendar	Escape the Vape	Quit Genius	Smoke Watchers	Aeris	Smokler
App classification		Tracker	Tracker	Tracker	Tracker	Tracker	Other	Tracker	Other
**Popularity**									
	Ratings on Apple App Store^a^	4.5 (1100)	4 (4)	0 (0)	3.4 (5)	4.3 (472)	0 (0)	2.3 (3)	0 (0)
	Ratings on Google Play Store^a^	4.5 (3000)	3.7 (248)	0 (0)	3.8 (6)	4.3 (2000)	3.7 (39)	3.9 (84)	4.7 (45)
	Number of Google Play Store downloads	100,000+	10,000+	100+	100+	100,000+	5000+	5000+	1000+
**MARS^b^ scores**									
	Engagement	4.1	3.8	3.4	3.53	3.1	2.9	2.6	2.3
	Functionality	4.9	4.67	4.75	4.67	4.12	3.89	4.5	4.88
	Aesthetics	4.83	4	4.5	4	3.83	4.17	3.67	3.5
	Information	3.14	3.1	2.86	3.1	3.21	2.64	2.36	2
	MARS total mean scores	4.24	3.89	3.88	3.82	3.57	3.4	3.28	3.17
**Adherence to Canadian clinical guidelines**									
	Ask about e-cigarette^c^ use status	✓	✓	✓	✓	✓	✓	✓	
	Advise user to quit	✓	✓	✓	✓	✓	✓	✓	✓
	Assess willingness to quit	✓	✓	✓		✓		✓	
	Assess nicotine dependence		✓			✓	✓		
	Assist—discuss the benefits of quitting	✓	✓	✓	✓	✓		✓	
	Assist—offer tools and resources for quitting	✓	✓	✓	✓	✓	✓	✓	✓
	Assist—enhance motivation to quit	✓	✓	✓	✓	✓	✓		
	Assist—explore doubts about quitting	✓							
	Assist—explore barriers to quitting	✓				✓		✓	
	Assist—affirm and encourage decision to quit	✓	✓	✓	✓	✓	✓	✓	
	Assist—form a quit plan							✓	
	Assist—discuss relapse prevention	✓		✓	✓		✓	✓	
	Assist—refer to a quitline							✓	
	Arrange for follow-up	✓		✓		✓			✓
	Adherence Index score (0-14)	12	8	9	7	10	7	10	3

^a^Number of stars out of 5 (number of reviews).

^b^MARS: Mobile App Rating Scale.

^c^e-cigarette: electronic cigarette.

### Data Extraction and Quality Assessment

Prior to assessing the full sample of apps, 3 raters (AK, EL, and SS) tested the MARS and Adherence Index with a randomly selected app. Results from the test app showed substantial agreement with minor differences (≤2 points) that were discussed and resolved. The remaining apps were rated independently by at least two authors. Intraclass correlation coefficient (ICC) estimates were computed to assess interrater reliability for the remaining apps. Results based on a mean rating (k=3), absolute agreement, and 2-way mixed effects model showed excellent agreement (ICC 0.92; 95% CI 0.78-0.98) [24].

## Results

### Overview

**Figure 1:**
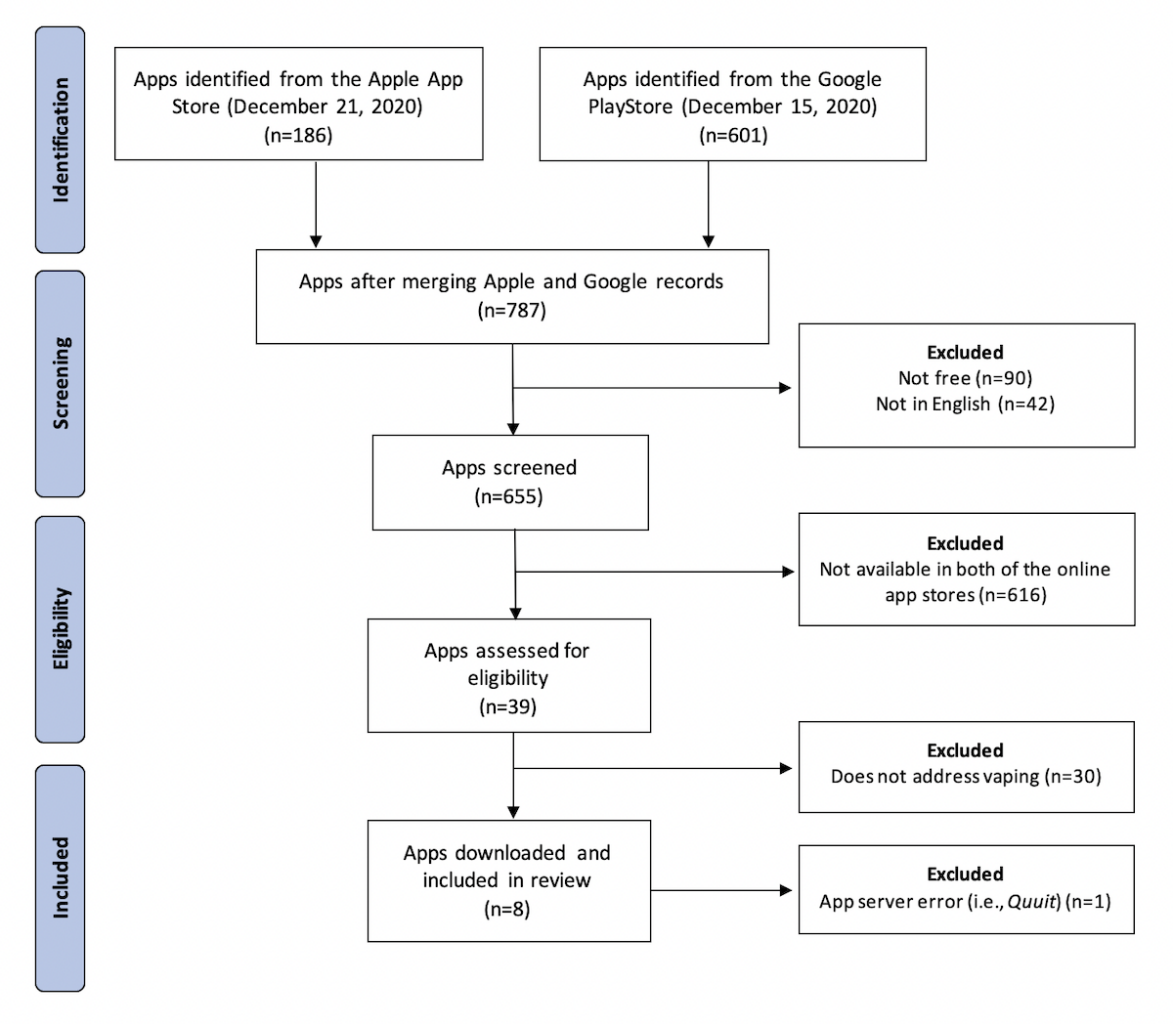
Flow diagram of the app selection process.

**Figure 2:**
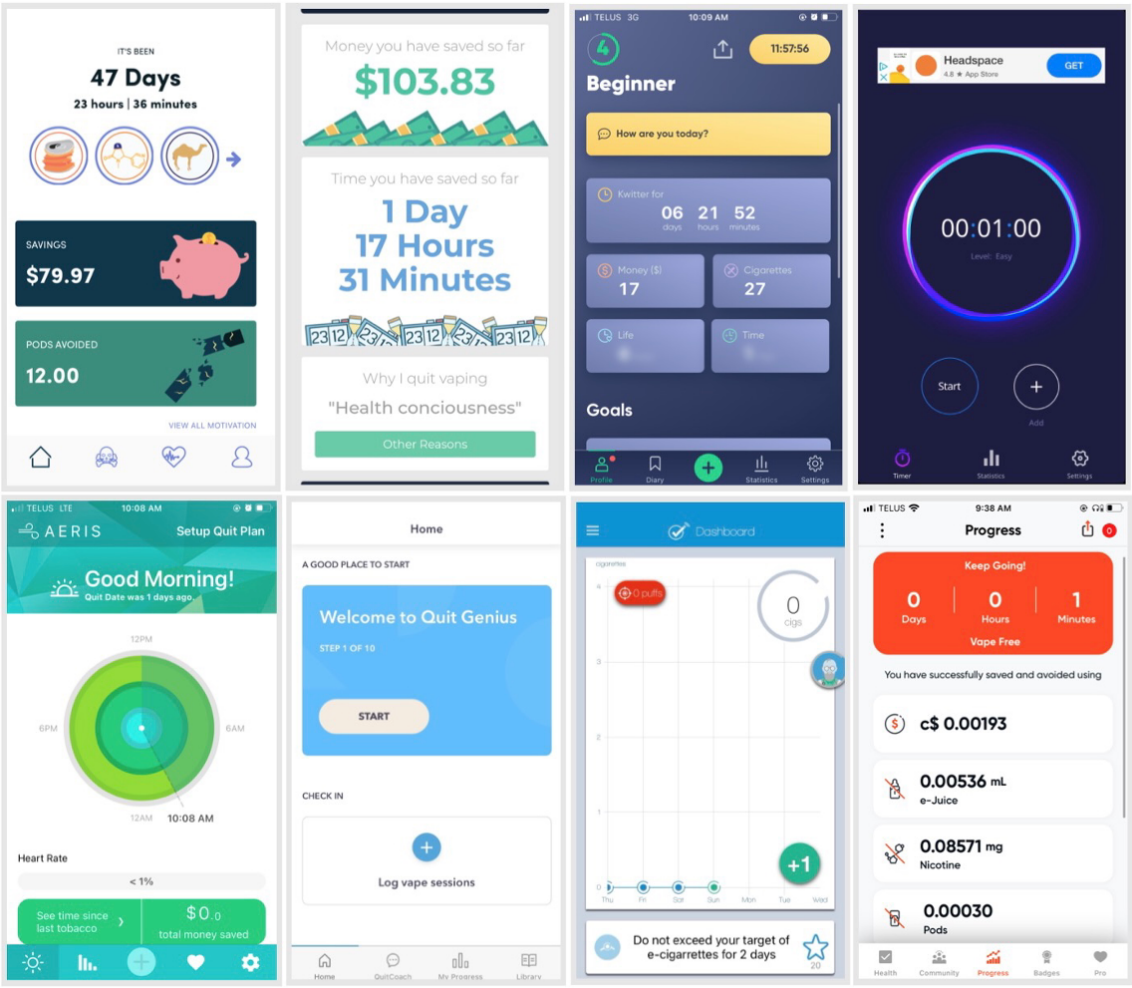
Visual overview of the 8 apps included in the analysis.

A total of 9 apps for vaping cessation were identified. Once the apps were downloaded, we discovered that 1 app (*Quuit*) was faulty and could not be examined past the sign-up page. Multiple attempts to reach the developers to resolve the issue were unsuccessful. At this point, we decided to exclude *Quuit*. The app selection process is shown in Figure 1. Figure 2 provides a visual overview of the remaining 8 apps included in the analysis. Characteristics and quality scores of each app are presented in Table 1.

### Assessment of Quality, Contents and Features, and Popularity Among Users

Among the 8 apps, the overall mean of the MARS total mean scores in Table 1 was 3.66, indicating *acceptable* quality. The mean rating for each of the 4 objective categories among the included apps is as follows: functionality was scored highest (4.54), followed by aesthetics (4.06) and engagement (3.22), while information had the lowest mean score (2.8). Cross-referencing the informational content on the apps with the current state of evidence on the health consequences of vaping [2], we found that some, but not all, informational text was supported by scientific evidence. A few other apps shared links to resources, which often pointed users to news headlines or anecdotal stories.

Of the 8 apps, 3 specifically targeted vaping cessation (*Escape the Vape*, *Quit Vaping Addiction Calendar*, and *Quit Vaping – For Good*) and 5 were focused on smoking cessation while also claiming to address vaping cessation. Although the remaining 5 apps claimed to address vaping, they were primarily developed for smoking cessation and many features were not fully adapted to vaping cessation. This is evident when users are asked to provide information on the number of cigarettes smoked and cost of a pack of cigarettes during the sign-up process. Even among the 3 apps developed primarily for vaping cessation, it was common to see the terms “vaping” and “smoking” used interchangeably. None of the apps asked users to enter information on basic demographics or vape flavor preferences. Some apps included valuable features such as a designated quit plan page that we later learned were available only with paid subscriptions ranging from US $5.99 to US $27.99. In total, 6 apps offered in-app purchases.

Of the included apps, 3 had a built-in community forum where users can anonymously share their experiences; 2 of these had features that allowed users to interact with others’ posts. However, 1 (*Smoke Watchers*) was entirely in French. A fourth app (*Kwit*) offered users the option to join a private support group on Facebook with a membership of 1800. Another app (*Quit Genius*) offered a community feature and access to a quit coach to premium users for US $27.99 per month.

Trackers, characterized as a combination of calculators and calendar apps, were the most common type of vaping cessation apps. As many as 6 of the 8 apps applied a tracker approach to vaping cessation, documenting money saved over time and number of days since quitting. A total of 4 tracker apps also provided a measure of health gains since quitting, while 2 presented estimations of “nicotine avoided” either in milligrams, number of pods, or both. These valuations, except for health gains, were based on information provided by users during the sign-up process. In addition to money spent on vaping and frequency of vaping, *Quit Vaping – For Good* asked users to indicate the type of vape device used, nicotine concentration preferred, number of pods in a pack, and amount of e-liquid content for each pod. It also tracked “total e-juice avoided.” All tracker apps, except *Aeris*, conferred awards corresponding to milestones achieved based on money saved and time vape free, as well as nicotine avoided and community engagement for those apps that had those features. Three of the tracker apps also kept a record of frequency and intensity of cravings that users can enter spontaneously within the app. Of these 3 apps, 2 presented users with a daily check-in: *Quit Genius* asked “Did you vape?” while *Kwit* asked users to provide a scale rating of their emotions and confidence in quitting.

Compared with the tracker apps, *Smoke Watchers* and *Smokler* implemented unique approaches. As the name indicates, *Smoke Watchers* connected vapers with watchers who may be vapers or nonvapers interested in supporting a vaper in their quitting process. Users can invite friends to be their watcher or select 1 from a community of watchers provided by the app. *Smoke Watchers* required vapers to connect Bluetooth-enabled vape devices directly to the app for real-time monitoring without an option for a user to manually enter their own data. Unfortunately, as none of the authors owned a vape device, this was not a feature that was fully explored. By contrast, *Smokler*’s primary approach to vaping cessation was implementing a vaping schedule. Users start a timer, then vape when the timer ends. There are varying levels of difficulty based on how long the timer is set for, and the interval between each vaping occasion increases according to the user’s preference.

All apps were developed commercially and most of them typically focused on abstinence with only 2 apps (*Smoke Watchers* and *Smokler*) offering users the option to modify their goals toward reduction or rationing. In terms of substances, nicotine was the primary focus in all 8 apps. *Quit Genius* was the only app linked to an efficacy trial published in the scientific literature [25], which concluded it to be a “superior treatment” compared with “very brief advice.” Notably, the authors disclosed that the study was funded by the company that developed *Quit Genius* and 8 of the 10 authors received a salary from or owned equity in the company.

Lastly, popularity among users was estimated using relative frequency of downloads, user ratings, and number of reviews. As shown in Table 1, the 3 most frequently downloaded apps (*Kwit*, *Quit Genius*, and *Quit Vaping – For Good*) were also those that were most highly rated and had the greatest number of user reviews, which suggests strong engagement among users. However, this association was not consistently present among the 5 remaining apps. To illustrate, *Aeris* (3.9 stars) had more user downloads and reviews compared with *Smokler*. However, *Smokler* (4.7 stars) had a higher user rating. In one of our searches using the search term *quit vaping*, *Aeris* was among the top 20 results in the Apple App Store, but *Smokler* was not. Interestingly, there were several apps with no reviews or user ratings that were included in the top 20 results. Overall, we found that a higher user rating was not associated with the order an app shows up in the list of results—a finding that is consistent with previous studies of a similar design [11,12].

### Adherence to Evidence-Based Practices

As shown in Table 1, the most commonly represented items on the Adherence Index were advising users to quit and assisting users by offering tools and resources for quitting. By contrast, the least common items were assisting users by exploring doubts about quitting, forming a quit plan, and referring them to a quitline. Each of these were only identified once among the apps included in the analysis. The *Kwit* app had the highest MARS total score, while also having the most Adherence Index items present. It also had the highest user rating based on data from the Apple App Store and the Google Play Store. Meanwhile, *Smokler* had the lowest MARS total score and also had the fewest Adherence Index items present. *Aeris* was the only app that recommended calling a quitline and the only one with a designated feature for setting up a quit plan.

## Discussion

### Principal Findings

This study identified 8 apps available for vaping cessation. Generally, these existing vaping cessation apps appear to employ similar approaches to smoking cessation apps that have been previously examined [11-15]. However, relative to smoking, apps that aid in quitting vaping are very low in number and are limited in features developed specifically for vaping cessation.

Although we found that the highest- and lowest-rated apps on the MARS tool received parallel scores on the Adherence Index, there was no association between the scores for the remaining apps. Similarly, there was no association between quality scores and app popularity based on user ratings and number of downloads. Previous studies with a comparable study design reported mixed results in this area: for example, Abroms and colleagues [12] concluded that user ratings were positively associated with Adherence Index scores, whereas Ubhi and team [13] found no such association. Consistent with findings in previous studies, we found that (1) the most common strategy employed to promote vaping cessation was tracking and monitoring [11,12,15]; (2) few apps included the behavior change techniques proposed to aid in quitting [13,14]; (3) the apps had little evidence-based informational content [13,14]; and (4) referrals to a quitline were absent [11-15]. As with these previous studies, we also found that the apps in our sample scored better in design and usability components over clinically relevant criteria. MARS items on quality of information and credibility of sources consistently received the lowest marks. These findings suggest that the development team may have been more focused on making the apps user-friendly than incorporating evidence-based content.

Overall, we found that using an app quality assessment tool and Adherence Index was a feasible approach to evaluating vaping cessation apps. Nonetheless, we discourage readers from interpreting these results to mean that some apps are more *effective* than others in promoting vaping cessation. We learned from this process that some apps are inherently different from others and it is not always appropriate, possible, or desirable to incorporate all of the items on the Adherence Index in any one app. The same issue arises in assessing apps based on the prevalence of behavior change techniques that have been positively associated with higher success rates for quitting smoking in *face-to-face* behavioral interventions [26]. This challenge was one that we encountered repeatedly in discussions about how items on the clinical guideline might be translated within the context of an app. For example, in this study, arranging for follow-up was translated to receiving push notifications from an app. However, several factors will influence the effectiveness of push notifications. Did the user enable notifications? Are there any consequences if a user ignores push notifications? What is the conversion rate between push notifications and interaction with the app? Do users experience desensitization to push notifications over time, thereby diminishing their usefulness? Similar questions were asked for other items on the guidelines and what they might look like in an app, although these were more straightforward compared with the example described here. Perhaps for this reason, an increasing number of studies are exploring the use of SMS text messaging or email services in promoting user engagement within an app [13,14] or as its own form of intervention [27]. Relative to vaping cessation, the best example of this is Truth Initiative’s *This is Quitting* program—a free and anonymous SMS text messaging service designed to help young people quit vaping, with preliminary results showing high levels of engagement among the target age group [7,28].

A reviewer of an earlier draft of this paper suggested that a qualitative analysis of user comments could shed more light on what features or characteristics users might find helpful and why. This is a potential way to identify features that future apps could incorporate to help support vaping cessation among users. The same reviewer inquired after possible approaches for addressing concurrent nicotine and cannabis use (or co-use) among users in light of emerging evidence of high rates of co-use and its implications for vaping cessation [29-32]. At the time of our review, we found no evidence of approaches for addressing co-use in our final sample of apps for vaping cessation. Certainly, apps for quitting cannabis use are available in both the Apple App Store and the Google Play Store and we encountered several of them during our searches. However, the degree to which these apps adhere to evidence-based practices warrants investigation. A review conducted in 2015 [33] that focused on the top 20 cannabis apps available online found that “no apps addressed abuse, addiction, or treatment.” As in ours, the 2015 review also showed that the majority of freely available apps were geared toward supporting recreational use.

Another possibility worth exploring is an “ecosystem of apps” to promote vaping cessation where users are free to choose the app (or set of apps) most appropriate for them based on their level of motivation, the stage of change they find themselves in, or simply personal preference. Ideally, this would be curated and maintained based on a similar process employed in this study and conducted by a group external to the app development team.

### Limitations

This study has several limitations. The evaluation of apps developed for health behavior change is a relatively new practice, and more guidance and resources are needed [34,35]. We acknowledge that app development occurs at a rapid pace and new apps are added regularly that were not available when we conducted our searches, and thus, would not be included here. Paid features offered as in-app purchases within apps that were free to download were also beyond the scope of this review. However, considering that free apps and features have been shown to increase accessibility and user engagement [36], our approach can be justified. Although our searches were conducted in the Canadian online app stores, it is likely that there is significant overlap in the online app market relative to the availability of vaping cessation apps. Finally, we were not able to assess the potential impact of these apps for different cultures and ethnicities.

### Strengths

This study also has noteworthy strengths. It is the first systematic analysis of apps for vaping cessation. As previous studies of a similar design have proven to be useful in areas such as alcohol and tobacco use [10-16], we recognize the value in assessing the quality and content of apps for vaping or e-cigarette use in informing research and practice moving forward. Based on our findings, we proposed several recommendations for the improvement of existing and future apps for supporting vaping cessation relative to the current state of evidence. Notably, we propose that researchers pay more attention to the unique qualities of vaping relative to smoking [8,23] and how these can be appropriately incorporated into apps focused on vaping cessation. Industry actors, such as the Apple App Store and the Google Play Store, might also have a role to play in improving accessibility to evidence-informed apps. For example, app stores could provide more transparency with regard to the algorithm behind their search results. One step further to this would be to explore the possibility of including clinical quality as a factor in the algorithm, in addition to the number of downloads and user ratings. The Apple App Store’s 2020 ban on recreational vaping apps [19] could be considered as a precedence for this kind of initiative. As vaping research evolves, there are plenty of opportunities to explore these recommendations and their potential for helping improve our understanding of how technology-based interventions, such as apps and SMS text messaging services, can be effective in helping users quit vaping. Finally, a key component to these recommendations is forthcoming research from 2 of the authors in this paper (AK and PS) offering clinical guidance for vaping cessation based on a systematic review. Beyond vaping research, this study also contributes to important and ongoing discussions over the role of mobile health apps in our health system, particularly in the field of substance use and addictions [37-40].

### Conclusions

Given the lack of vaping cessation interventions at a time when they are urgently needed, vaping cessation apps are potentially valuable tools. Therefore, it is recommended that these apps apply evidence-based practices and undergo rigorous evaluations that can assess their quality, contents and features, and popularity among users. Through this process, we can improve our understanding of how apps can be effective in helping users quit vaping.

## Abbreviations

e-cigarette: electronic cigarette
ICC: intraclass correlation coefficient
MARS: Mobile App Rating Scale
